# Directed Evolution of *Clostridium thermocellum* β-Glucosidase A Towards Enhanced Thermostability

**DOI:** 10.3390/ijms20194701

**Published:** 2019-09-23

**Authors:** Shahar Yoav, Johanna Stern, Orly Salama-Alber, Felix Frolow, Michael Anbar, Alon Karpol, Yitzhak Hadar, Ely Morag, Edward A. Bayer

**Affiliations:** 1 Department of Plant Pathology and Microbiology, Robert H. Smith Faculty of Agriculture, Food and Environment, the Advanced School for Environmental Studies, The Hebrew University of Jerusalem, Rehovot 76100, Israel; shaharyoav@gmail.com (S.Y.); hadar@agri.huji.ac.il (Y.H.); 2 Department of Biomolecular Sciences, The Weizmann Institute of Science, Rehovot 7610001, Israel; jostern@rcip.co.il (J.S.); orlysala@gmail.com (O.S.-A.); michaelanbar@gmail.com (M.A.); elymorag11@gmail.com (E.M.); 3 Department of Molecular Microbiology and Biotechnology, Tel Aviv University, Tel Aviv 6997801, Israel; hanbayer@gmail.com; 4 CelDezyner, 2 Bergman St, Tamar Science Park, Rehovot 7670504, Israel; alonkarpol@gmail.com

**Keywords:** Cellulase, random mutagenesis, cellulose degradation, structural analysis

## Abstract

β-Glucosidases are key enzymes in the process of cellulose utilization. It is the last enzyme in the cellulose hydrolysis chain, which converts cellobiose to glucose. Since cellobiose is known to have a feedback inhibitory effect on a variety of cellulases, β-glucosidase can prevent this inhibition by hydrolyzing cellobiose to non-inhibitory glucose. While the optimal temperature of the *Clostridium thermocellum* cellulosome is 70 °C, *C. thermocellum* β-glucosidase A is almost inactive at such high temperatures. Thus, in the current study, a random mutagenesis directed evolutionary approach was conducted to produce a thermostable mutant with K_cat_ and K_m_, similar to those of the wild-type enzyme. The resultant mutant contained two mutations, A17S and K268N, but only the former was found to affect thermostability, whereby the inflection temperature (T_i_) was increased by 6.4 °C. A17 is located near the central cavity of the native enzyme. Interestingly, multiple alignments revealed that position 17 is relatively conserved, whereby alanine is replaced only by serine. Upon the addition of the thermostable mutant to the *C. thermocellum* secretome for subsequent hydrolysis of microcrystalline cellulose at 70 °C, a higher soluble glucose yield (243%) was obtained compared to the activity of the secretome supplemented with the wild-type enzyme.

## 1. Introduction

Cellulose, the major polymer in the plant cell wall, is the most abundant organic resource on Earth, and cellulosic is a primary feedstock for the production of ethanol-based biofuels. Cellulose is a highly crystalline homopolymer composed of individual chains of glucose, which form a planar structure, reinforced by inter and intra-chain hydrogen bond interactions [1]. Depending on the source, each chain contains from 100 to more than 10,000 glucose units, with the disaccharide cellobiose (composed of two glucose units oriented at 180° along the chain axis) being its smallest repetitive unit [2]. In order to utilize cellulose as a resource for biofuel production, the chain must first be enzymatically hydrolyzed into its primary monomeric glucose units. The glucose is then used as a carbon source for alcoholic fermentation to produce bioethanol. Alternative fermentation processes can also be used for the production of various other biochemicals such as butanol, acetone, lactic acid, succinic acid and more. Thus, efficient enzymatic hydrolysis of the cellulose is crucial for increasing the cost-effectiveness of the bioethanol and biochemical production process [3,4].

In the plant cell wall, cellulose is encompassed by the hemicelluloses and lignin, which together create a chemically complex and recalcitrant structure [5,6]. The plant cell wall structure and the rigid nature of cellulose itself render the cellulose degradation process extremely difficult. For efficient degradation, a diverse set of plant cell wall-degrading enzymes is therefore required [7,8]. Cellulose hydrolysis is mediated by three major types of enzymes: endoglucanases, exoglucanases and β-glucosidases that work in synergy [9]. Endoglucanases can cleave the cellulosic chains in the middle, creating reducing and non-reducing ends. Exoglucanases hydrolyze the cellulosic chains from the newly formed chain ends in a “processive” (sequential) manner, leading to the formation of the soluble disaccharide cellobiose. Finally, β-glucosidases are capable of cleaving cellobiose into soluble glucose units. Cellobiose is known to serve as a strong feedback inhibitor (mainly for exoglucanases) [10], thus highlighting the significance of the β-glucosidases not only in providing the end product (glucose) but also in preventing feedback inhibition. For example, near-complete inhibition of the cellulosome of *Clostridium thermocellum* was observed at a concentration of only 2% cellobiose [11,12]. The addition of its native β-glucosidase A (BglA) was able to relieve inhibition, and thus to enhance the rate and degree of solubilization of crystalline cellulose [13,14]. The important role of BglA was also demonstrated by manipulating *C. thermocellum* 27405 to overexpress the BglA gene in vivo. The resultant strain demonstrated increased total cellulolytic activity during growth [15].

Extensive efforts have been made during the last decades for the development and assembly of efficient cellulolytic enzymatic cocktails. However, cellulose degradation is still not efficient enough to be cost effective [3,16]. One of the key bottlenecks for achieving cost-effective degradation of plant cell wall biomass is the requirement for large amounts of cellulases (about 100–200 g of cellulase per gallon of cellulosic ethanol) [17]. In this context, thermostable enzymes are gaining wide interest in the industry, since they are better suited for harsh process conditions, such as those used for the bioethanol production [18]. Thermostability of enzymes can be increased by genetic modification. Increasing the thermostability of an enzyme, while retaining its activity, is thought to enhance its overall performance, especially for the extended time periods necessary for degradation of cellulosic substrates [19,20]. Moreover, thermostable enzymes can be recycled more efficiently, thereby lowering overall production costs [21,22]. Finally, thermostable processes can reduce contamination [23]. Taken together, engineering thermostable enzymes is important to achieve the relatively low-cost biodegradation of biomass for the production of cellulosic ethanol [24]. Indeed, a wide range of bacterial or fungal cellulases was subjected to genetic modification to increase their thermostability [24,25,26,27,28].

*C. thermocellum* is a thermophilic bacterium, and its cellulosome is considered to be one of the most efficient natural systems for cellulose conversion [29]. Our group and others have previously reported the design of highly thermostable mutants derived from *C. thermocellum* cellulolytic enzymes, such as the endoglucanase Cel8A [30,31,32] and the exoglucanase Cel48S [33], which have proved to be stable at very high temperature ranges (around 80 °C). In nature, *C. thermocellum* utilizes cell-surface-bound cellulosomes to hydrolyze the cellulose into soluble cellobiose and other cellodextrins, which are then actively transported into the cells and hydrolyzed to glucose by periplasmic β-glucosidase [10,34]. The maximal cellulose degradation capacity mediated by the *C. thermocellum* cellulosome could be achieved at 70 °C [35]. However, the results reported here demonstrate inactivation of the recombinant *C. thermocellum* BglA (Clo1313_2020) at such high temperatures. Thus, and due to the important role of β-glucosidases, enhancing the thermostability of *C. thermocellum* BglA is of great significance and was the goal of the current study.

Directed evolution, which consists of random mutagenesis and high-throughput screening approaches, is a powerful technique which does not require prior functional, structural, or mechanistic knowledge. Only suitable and effective screening strategies for the desired activity are required [36,37]. The chromogenic product released from *p*-nitrophenyl-β-d-1,4-glucopyranoside (*p*NPG, an analogue of the natural substrate of β-glucosidase: cellobiose), enables efficient and rapid screening. Indeed, directed evolution methodologies have already been used in the past for enhanced thermostability of β-glucosidases from other (mostly mesophilic) organisms [36,38,39], which resulted in the creation of mutants stable at temperature ranges of 50–60 °C (the natural wild-type range of *C. thermocellum* recombinant enzymes). In the current study, we used the directed evolution strategy based on the substrate analogue *p*NPG, in order to create and reveal thermostable mutants of *C. thermocellum* BglA. The functionality of this mutant was also validated under near-natural conditions, namely by examining the contribution of the thermostable BglA to the hydrolyses of microcrystalline cellulose by *C. thermocellum* cellulase mixtures.

## 2. Results

### 2.1. Construction and Screening of C. Thermocellum BglA Clones Library

In order to generate thermostable mutants of *C. thermocellum* BglA, in vitro directed evolution was applied on the full-length open reading frame (ORF) of the Clo1313_2020 gene. High numbers of mutation events per clone enable broad screening, but, in contrast, too many mutation events per clone might mask the desired mutation events. Here, a frequency leading to ~20% active clones was chosen. Mutation frequency is determined by two parameters: the template amount and the number of polymerase reaction cycles. Hence, by using different template amounts and different thermal cycle numbers, appropriate mini-libraries were created. The amount of 100 ng DNA template and 23 PCR thermal cycles led to 23% active colonies, which were used to create a library of ~40,000 clones. Sequencing eight active and non-active clones revealed an average of three and seven mutation events per active and non-active clones, respectively. In the next step, the library was screened for thermostable clones. About 8000 clones were screened, revealing 40 thermostable (red) clones.

### 2.2. Characterization of the Thermostable Mutants

In order to verify and quantify the thermostability of the detected clones, their residual activity after heat shock was calculated (residual activity was defined as the activity of the heat-shocked lysate × 100/activity of non-heated lysate). The residual activities of the two most thermostable clones were 149% and 140% higher than the wild type. The most thermostable clone was sequenced, revealing one silent mutation and two mutation events: A17S and K268N (herein referred to as Mut 1). The second thermostable clone revealed a single mutation event: S39T (referred here as Mut 2).

**Figure 1:**
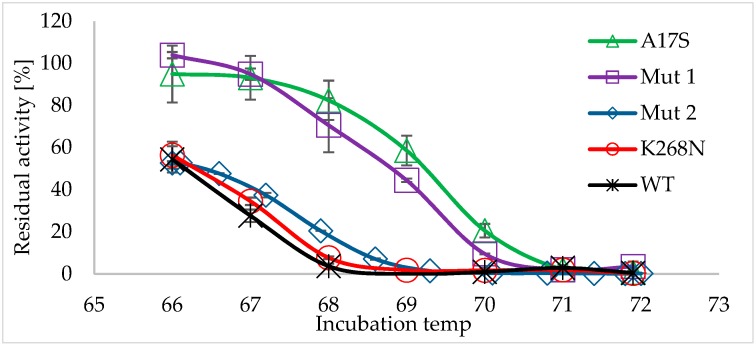
Thermostability of the various *C. thermocellum* BglA mutants. Wild-type (WT) *C. thermocellum* BglA and the different mutants were incubated at 66–72 °C for 1 h, followed by activity assay (using *p*-nitrophenyl-β-d-1,4-glucopyranoside [*p*NPG], an analogue of the natural β-glucosidase substrate). Residual activity of the different mutants (defined as the activity of the heat-shocked enzyme × 100/activity of the non-heated enzyme) was calculated. Mut 1 and A17S demonstrated similar thermostability.

For further characterization, the two thermostable mutants and the wild-type BglA enzyme were recombinantly expressed in *E. coli* and purified. Purified enzymes were subjected to heat-shock treatment (66–72 °C, 1 h) and residual activities were calculated (Figure 1). Indeed, both mutants were more thermostable than the wild-type enzyme, with Mut 1 being more thermostable than Mut 2. Mut 1 lost only ~40% of its activity (residual activity ~60%) after heat-shock at 68.4 °C. Under the same conditions, Mut 2 lost ~90% of its activity, and the wild-type BglA totally lost its activity. After heat-shock at 70 °C, both the wild-type and Mut 2 enzymes totally lost their activity, while Mut 1 still exhibited residual activity of ~9.5%.

The kinetic parameters of the wild type and Mut 1 were measured (Table 1). Mut 1 revealed relatively similar and only slightly higher catalytic efficiency (K_cat_/K_m_), with no statistical differences between the K_cat_ and K_m_ values compared to the wild type enzyme. The effect of each mutation event (namely A17S and K268N) on the thermostability of Mut 1 was examined. For this purpose, two recombinant enzymes were constructed and purified, one containing only the A17S mutation, and the other only K268N. The enzymes were subjected to heat-shock (66–72 °C, 1 h), and the residual activities were calculated (Figure 1). A17S demonstrated similar thermostability to Mut 1, indicating the major contribution of this substitution to the thermostability. In contrast, K268S did not demonstrate improvement in its thermostability (compared to that of the wild type), indicating that the improved thermostability derived only from the A17S mutation event. Using site-direct mutagenesis, A17 was substituted with other polar amino acids, namely, threonine, glutamine and asparagine. The resultant enzymes (A17T, A17Q and A17N) demonstrated much lower activity and thermostability, compared to the wild-type enzyme (Figure S1).

**Figure 2:**
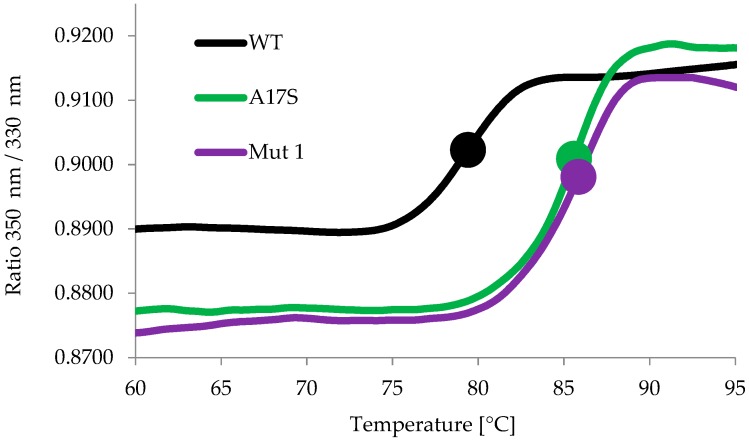
Inflection temperatures (T_i_) of wild-type (WT) *C. thermocellum* BglA, Mut 1 and A17S. The inflection temperatures were measured using a NanoTemper Tycho NT.6 instrument as described in the Methods section.

The inflection temperatures (T_i_) of the wild-type, Mut 1 and A17S enzymes were measured using a NanoTemper Tycho NT.6 instrument. This assay is based on the changes in the intrinsic fluorescence from the aromatic amino acid residues tryptophan and tyrosine (measured at 350 nm and 330 nm). During the assay, the temperature of the protein solution is ramped from 35 °C to 95 °C for a 3 min period, accompanied by continuous measurement of the fluorescence. Changes in the fluorescence signal indicate transitions in the folding state of a protein. The midpoint temperature at which a transition occurs is called the inflection temperature (T_i_) [40,41,42]. The T_i_ of wild-type BglA, Mut 1 and A17S were 79.3 ± 0.08, 85.7 ± 0.15 and 85.7 ± 0.16 °C, respectively, demonstrating an increase of ~6.4 °C in the T_i_ (Figure 2). The effect of cellobiose (the natural substrate of β-glucosidases) added to the reaction mixture (at 1 mM) on the T_i_ was also tested. The results were similar (80 ± 0.48 °C, 85.9 ± 0.53 °C and 85.8 ± 0.1 °C for the wild-type, Mut 1 and A17S, respectively), indicating no stability effect in the presence of cellobiose.

**Figure 3:**
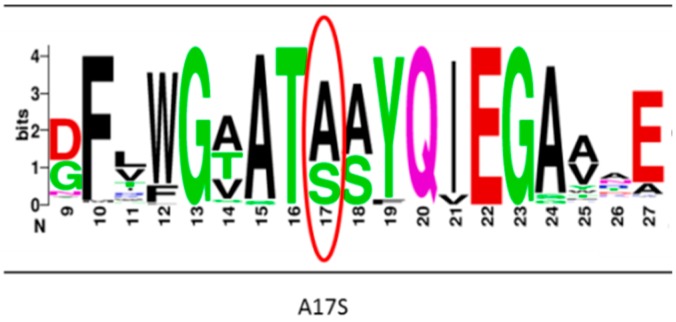
Amino acid frequencies in the residues surrounding the A17S mutation event. The relevant amino acid sequence of *C. thermocellum* BglA was BLASTed against the NCBI nonredundant protein database. The top 1000 hits were used to create the distribution scheme using WEBLOGO. Position 17 is marked by the red circle.

The amino acid sequence of *C. thermocellum* BglA was BLASTed against the NCBI nonredundant protein database. BLAST results for the residues adjacent to the A17S mutation event are represented schematically by the diagrams of amino acids frequencies in Figure 3. The residues near position 17 were found to be relatively conserved. Interestingly, the only amino acid replacing the alanine in position 17 in the different homologues was serine.

### 2.3. Structural Aspects of BglA

The crystal structure of wild-type *C. thermocellum* BglA was recently determined in our lab (PDB code 5OGZ, Table S1). BglA adopts the expected (β/α)_8_ TIM barrel fold, typically observed for clan-A β-glucosidases, with two active-site glutamates, Glu166 on strand β4 and Glu355 on strand β7, presumed to act as catalytic acid/base and nucleophile, respectively. Structure alignment of the Bg1A structure with those of four other family-1 β-glucosidases revealed that the glutamate residues are positioned very similarly with respect to the aligned structures. In addition, the distance between their Cδ atoms is 5.33 Å, consistent with the properties of a retaining β-glycosidase. These highly conserved motifs are responsible for substrate binding and enzymatic hydrolysis of the glycosidic bond within the active site.

**Figure 4:**
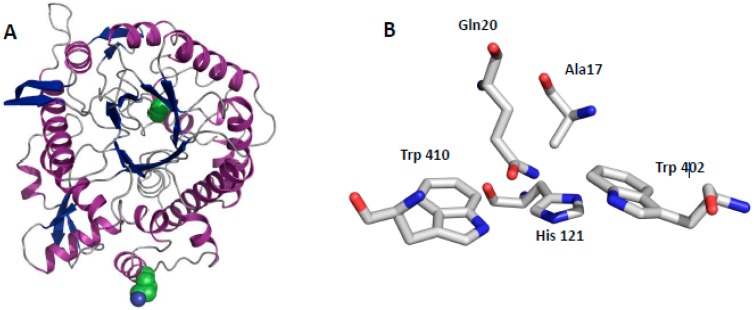
Structural analysis of *C. thermocellum* BglA. (**A**) Crystal structure of *C. thermocellum* BglA. α-Helices are colored purple. β-sheets are colored blue. Ala17 and Lys268 (the two mutated positions in Mut 1) are displayed by spheres. (**B**) Ala17 and its adjacent residues. Carbon atoms are shown in white, oxygen atoms in red and nitrogen atoms in blue. Analysis was performed using PyMol software.

Figure 4A shows the position of A17, which is located in a loop near the central cavity of the enzyme. The nearby coding region of K268, however, which is located in an outer α-helix, was relatively non-conserved (data not shown).

### 2.4. Advantage of Thermostable BglA in the Cellulose Hydrolysis Process

**Figure 5:**
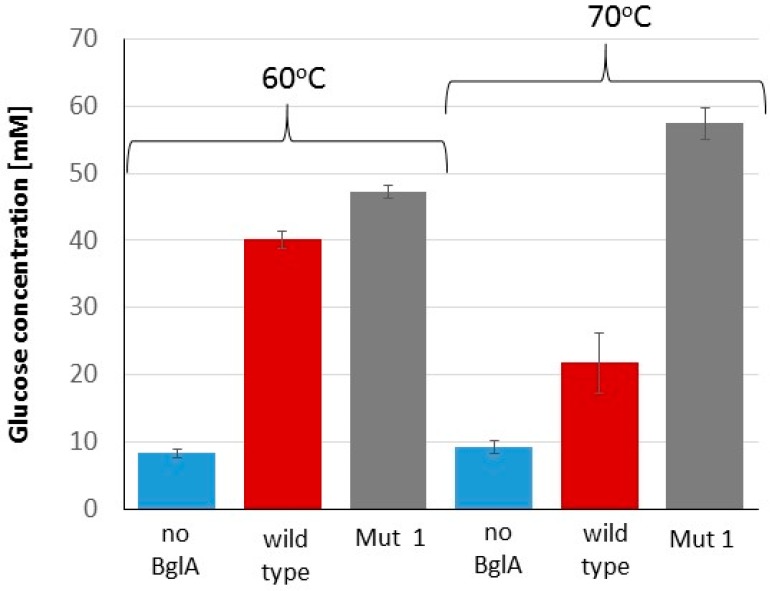
Hydrolysis of microcrystalline cellulose by the *C. thermocellum* secretome. Solutions containing the C*. thermocellum* secretome were applied on Avicel with or without the addition of BglA (wild-type or mutant), followed by incubation at either 60 or 70 °C for 48 h. The concentration of the released soluble glucose was measured by HPLC. Mut 1 showed higher glucose yields at both temperatures, with significant advantage at 70 °C. The experiment was conducted in triplicate. Bars indicate standard deviation.

The contribution of thermostable BglA (Mut 1) to the cellulose hydrolysis process was examined. For this purpose, microcrystalline cellulose (Avicel) was hydrolyzed by the *C. thermocellum* secretome, supplemented with either Mut 1 or wild-type BglA at 60 and 70 °C, and the concentration of the released soluble glucose was measured (Figure 5). Final glucose concentrations in the Mut 1 samples were higher than those of the wild-type samples at both temperatures. However, this advantage was much higher while working at 70 °C (57.4 mM in Mut 1 versus 21.7 mM in the wild-type samples), and lower while working at 60 °C (47.1 mM in Mut 1 versus 40.1 mM in the wild-type).

In addition, Mut 1 was integrated in our lab into a thermostable designer cellulosome [43]. To do so, a plasmid was created containing clone 1 attached to the gene segment coding for the dockerin module from *Clostridium clariflavum*. The expressed recombinant enzyme was successfully integrated into an artificial thermostable designer scaffoldin, which also contained appropriate dockerin-bearing thermostable *Clostridium thermocellum* mutants of both exoglucanase Cel48S and endoglucanase Cel8A. The resulting thermostable designer cellulosome exhibited a 1.7-fold enhancement in cellulose degradation (compared to the action of conventional designer cellulosomes that contain the respective wild-type enzymes). The results were published by Moraïs et al. in 2016 [43].

## 3. Discussion

Developing thermostable and highly active cellulase preparations is critical for achieving cost-effective enzymatic deconstruction of cellulosic biomass [24]. In this report, directed evolution was conducted on the β-glucosidase A gene of the thermophilic bacterium, *C. thermocellum*, to produce a potent thermostable mutant (Mut 1), which contained two mutations: Alanine at position 17 was substituted with serine, and lysine at position 258 was substituted with asparagine. However, only the A17S mutation was found to be responsible for the observed thermostability. The residues near position 17 were found to be conserved among the top 1000 homologous sequences of *C. thermocellum* β-glucosidase A. The observed conservation can indicate the important role of this position and on the importance of alanine 17 to the functionality of the enzyme [44,45]. The fact that serine was the only amino acid replacing alanine in position 17 in the different homologues is fully consistent with our results, in which the alanine-to-serine mutation produced a functional and thermostable mutant enzyme. Indeed, substitution of A17 with threonine, glutamine and asparagine resulted in almost inactive enzymes. According to Daniel et al. (1996), the enhanced stability of proteins can be achieved by an additional stabilizing force which is equivalent to only a few weak interactions [31,46]. Indeed, single mutation events were found to increase the thermostability of various cellulases [31,36,47]. In the case of *C. thermocellum* BglA, substitution of serine for alanine would likely result in hydrogen bonding with His 121 (Figure 4B), both of which are located near the active site. The newly created hydrogen bond would presumably help stabilize the enzyme. Our results also demonstrate that a single point mutation can increase the thermostability of the already naturally thermostable *C. thermocellum* BglA, thereby increasing its T_i_ by 6.4 °C.

Mut 1 showed increased activity over the wild-type enzyme, with a much higher effect at 70 °C versus 60 °C (the optimum growth temperature for *C. thermocellum*). These results, together with the fact that Mut 1 has similar K_cat_ and K_m_, indicate that the mutation affects mainly the thermostability, rather than the activity of the enzyme. Directed evolution conducted on the mesophilic bacterium *Paenibacillus polymyxa* BglA revealed a more thermostable mutant containing the same A17S mutation event [36]. This mutation event increased the half-life of thermoinactivation by 11-fold, when applied at 50 °C. In addition, the authors obtained a lower K_m_ and a higher K_cat_, resulting in doubling the catalytic efficiency. Considering the crystal structure of *P. polymyxa* BglA, a possible explanation for those effects was suggested [36]. The alanine at position 17 occurred in the internal cavity, buried among Gln20, His121, Trp398 and Trp406, near the active site. It was suggested that the alanine-to-serine substitution increased the residue volume, which in turn was assumed to be important for enzyme thermostability [48]. In addition, the proximity of the newly substituted polar residue (serine) to the above-mentioned amino acids, all of which participate in the ligand binding, was suggested to increase the binding affinity [49]. The crystal structure of *C. thermocellum* BglA revealed a similar structure (Figure 4B) to that of *P. polymyxa* BglA, with the Ala17 buried in the internal cavity among Gln20, His121, Trp402 and Trp410. Thus, a very similar mechanism may well be valid for *C. thermocellum* BglA although no significant effect on K_m_ was measured.

Chromogenic substrates are often used for the screening of enzyme activity, since they enable rapid visual detection of the desired phenotype. However, improved hydrolysis of the synthetic chromogenic substrate does not necessarily correlate with that of the natural substrate of the enzyme [38,50,51]. Thus, a verification step of the enzymatic activity on the natural substrate is required following screening on substrate analogues. Thus, the contribution of Mut 1 to cellulose hydrolysis was tested in this study, thereby revealing its advantage at higher temperatures.

In nature, the assembly of the catalytic units of *C. thermocellum* into cellulosomes containing cellulose-binding modules (CBMs) resulted in the formation of higher local cellobiose concentrations at particular sites. However, *C. thermocellum* BglA does not possess a CBM module, and is not targeted towards the increased local cellobiose concentration. Instead, cellulosome-generated cellobiose is transported directly into the cell [34], and hydrolyzed to glucose by periplasmic β-glucosidases. In cell-free enzymatic systems, such as that reported here, removal of inhibitory cellobiose can be performed by adding BglA to the assay. Thus, targeting the recombinant BglA to the increased local cellobiose concentration might improve hydrolysis efficiency. In a former study conducted by our lab, we reported the design of a recombinant form of the wild-type *C. thermocellum* BglA, which possessed the ability to directly bind to the cellulosome via cohesin-dockerin interaction [52]. Integration of BglA into the *C. thermocellum* cellulosome led to higher degradation levels of microcrystalline cellulose and pretreated switchgrass, compared to cellulosomes supplemented with the soluble wild-type form of the enzyme. By using the same technique, the thermostable Mut 1 was incorporated into thermostable designer cellulosomes, which now demonstrated a 1.7-fold enhancement in cellulose degradation, compared to a non-thermostabilized designer cellulosome preparation [43]. These results further emphasize the advantage of thermostable mutants for improving lignocellulosic biomass conversion.

Several approaches can be applied to design thermostable cellulases. In the current study, the directed evolution approach was used for the improvement of *C. thermocellum* BglA. This strategy is based on random mutagenesis and sequential screening rather than a rational hypothesis-based approach. This powerful method does not require preliminary knowledge about the structure of the enzyme, and can reveal mutations that would not be revealed by knowledge-dependent approaches. However, in the future, the recently solved crystal structure of *C. thermocellum* BglA (Table S1) can be used for rational design, in order to further improve the thermostability and activity of Mut 1. [53].

Overall, the current study demonstrates that natural thermostable cellulases can be further improved. Exposing the “hidden” potential of plant cell wall-degrading enzymes is thus an important step towards cost-effective conversion of plant biomass into bioethanol or other biochemicals.

## 4. Materials and Methods

### 4.1. Random Mutagenesis and Library Construction

A library of *C. thermocellum* BglA mutant clones was created as previously described [30,31] with minor modifications. Different quantities (20, 100 or 400 ng) of the *C. thermocellum* BglA open reading frame Clo1313_2020, cloned in a pET28a plasmid, were used as a template for error prone PCR according to the manufacturer’s instructions, using Gene-Morph II Random Mutagenesis Kit (Stratagene, La Jolla, CA, USA). Thermal cycling parameters were 95 °C for 2 min followed by 18, 23 or 27 cycles of 95 °C for 1 min, 55 °C for 45 s and 72 °C for 2 min, followed by a final step of 72 °C for 10 min. T7 promoter primer and T7 terminator primer were used for amplification. The resulting PCR products were applied on 0.75% agarose gel. Extracted bands were treated with DpnI and diluted 100 times. The solution was then used as a template for sequential PCR reaction using ReadyMix^™^ Taq PCR Reaction Mix (Sigma-Aldrich, Rehovot, Israel) with primers: CAGTC**CATGGC**AAAGATAAC (NcoI restriction site) and CACG**CTCGAG**GAAACCGTTGTTTTTGATTAC (XhoI restriction site). The thermal cycling parameters followed the manufacturer’s instruction, with an annealing temperature of 55 °C and elongation time of 2 min. The amplified products were purified, restricted with NcoI and XhoI according to the manufacturer’s instructions and ligated to the pET28a-based plasmid treated with NcoI/XhoI and shrimp alkaline phosphatase (SAP). The ligated plasmids were treated with SacI in order to remove unrestricted vector. (New England Biolabs, UK enzymes were used in the restriction and ligation process). Plasmids were then electrotransformed into *E. coli* XL1 electro-competent cells and purified using a miniprep kit (QIAprep Spin Miniprep Kit, Qiagen, Redwood City, CA, USA), creating minilibraries. The minilibraries were transformed into *E. coli* BL21 competent cells and plated on LB plates, containing 1.5% agar, 4 µM isopropyl β-d-1-thiogalactopyranoside (IPTG) and 50 µg/mL kanamycin. The plates were incubated overnight at 37 °C. A solution of 25 mM citrate buffer, pH = 6.1, containing 0.75% agar, was then boiled, cooled to 45 °C and supplemented with Magenta GlcA (5-Bromo-6-chloro-3-indolyl β-d-glucuronide cyclohexylammonium salt, Sigma Aldrich) to a final concentration of 0.02%, and applied onto the plates, creating an additional layer. The plates were dried for 1 h at room temperature and then incubated at 60 °C for 1.5 h (until red colonies appeared). The percentage of the red colonies was calculated and the parameters leading to 20–30% active clones were further used to increase library.

### 4.2. Screening for Thermostable Clones

Library screening was performed as detailed in the previous section with two additional steps: Plasmids were transformed into *E. coli* BL21 competent cells and plated on LB plates containing 1.5% agar, 4 µM IPTG and 50 µg/mL kanamycin. The plates were incubated overnight at 37 °C. They were then replicated on fresh LB agar plates using silk snippets, heat-shocked at 70 °C for 50 min and cooled at 4 °C. A layer of 25 mM citrate buffer, pH 6.1, 0.75% agar and 0.02% Magenta GlcA, was added to the plates (as detailed above), which were dried for 1 h at room temperature and then incubated at 60 °C for 1.5 h. The replicates were used to purify the plasmids of selected red colonies, and selected clones were sequenced. A plate containing wild-type *C. thermocellum* BglA was used as a control.

### 4.3. Residual Activity of Overexpressing BglA Colonies

Colonies overexpressing thermostable mutants (as indicated by the appearance of red color) were grown on liquid LB medium (0.5 mL), containing 0.1 mM IPTG and 50 µg/mL kanamycin, overnight at 37 °C in 96-deep-well plates. In order to extract the proteins, each well was supplemented with 20 μL of Popculture (Novagene, Darmstadt, Germany), DNaseI and lysozyme, and the plate was incubated at 37 °C for 20 min. The lysate was diluted 30 times in 50 mM citrate buffer, pH 6.1. Diluted lysate (100 µl) was incubated at 66 °C for 75 min and cooled on ice. Then, 15 μL of heated and non-heated samples were added to 1 mM *p*-nitrophenyl-β-d-glucopyranoside (*p*NPG, Sigma Aldrich, St. Louis, MO, USA) solution and incubated for 45 min at 60 °C. The reaction was terminated by adding 85 µl of 1 M carbonate buffer, pH 9.5, and optical densities of the samples were measured at a wavelength of 405 nm. Residual activity was calculated by comparing the activity of the heated versus non-heated samples.

### 4.4. Protein Expression and Purification

The resulting *C. thermocellum* BglA mutants and wild-type enzymes were produced by expression of relevant plasmids into *E. coli* BL21 (lDE3) pLysS cells. The proteins were extracted and purified on an Ni-nitrilotriacetic acid (NTA) column (Qiagen, Hilden, Germany), as reported earlier [30]. Purity of the recombinant proteins was assessed by SDS-PAGE on 12% acrylamide gels, and fractions, containing the pure recombinant protein, were pooled and concentrated using AmiconUltra 15 mL 50,000 MWCO concentrators (Millipore, Bedford, MA, USA). Protein concentration was estimated from the absorbance at 280 nm, based on the known amino acid composition of the protein, using the Protparam tool (http://www.expasy.org/tools/protparam.html). Proteins were stored in 50% (*v*/*v*) glycerol at −20 °C.

### 4.5. Stability Assay

Solutions of 50 mM citrate buffer, pH 6, containing 7 µg/mL of the recombinant enzymes, were incubated at 66–72 °C for 1 h and then cooled on ice. Heated and non-heated samples were diluted to a final enzymatic concentration of 1.05 µg/mL in a solution of 50 mM citrate buffer, pH 6.1, containing 1 mM *p*NPG, incubated for 10 min at 60 °C, cooled on ice, and supplemented with equal amounts of 1 M carbonate buffer, pH 9.5. Optical densities of the samples were measured at a wavelength of 405 nm. Residual activity was calculated by comparing the activity of the heated versus non-heated enzymes.

### 4.6. Kinetic Parameters Measurements

A solution of 50 mM citrate buffer, pH 6.1, containing 0–25 mM *p*NPG, was supplemented with a concentration of 13 nM enzyme (wild-type or mutant) and incubated at 60 °C for 8 min in a preheated 96-well plate, accompanied by continued measurements at OD_405_. The concentrations of end product (*p*-nitrophenol) were calculated using known concentrations of *p*-nitrophenol. Kinetic parameters were calculated by nonlinear fit using the GraphPadPrism software (GraphPad Software, Inc., San Diego, CA, USA)

### 4.7. Sequence Analysis

The protein sequence of *C. thermocellum* BglA (ADU75064.1) was BLASTed against the NCBI non-redundant protein database. The top 1000 hits with *E*-value < 0.001 were further aligned. Frequency of amino acids was visualized using WEBLOGO version 2.8.2.

### 4.8. Purification of the C. Thermocellum Secretome

*C. thermocellum* DSM1313 was grown on GS-2 medium (0.5 g/L K_2_HPO_4_, 0.5 g/L MgCl_2_·6H_2_O, 0.5 g/L KH_2_PO_4_, 1.3 g/L (NH_4_)_2_SO_4_, 0.002 g/L resazurin, 10.5 g/L MOPS buffer, 5 g/L yeast extract, 1.25 mg/L iron(II) sulfate and 0.5 mM CaCl_2_, adjusted with 10 M NaOH to a final pH of 7.2) with 0.5% microcrystalline cellulose (Avicel, Sigma Aldrich, St. Louis, MO, USA) in batch culture. Nitrogen flushing was used to achieve anaerobic conditions. After 48 h of incubation at 60 °C, growth medium was centrifuged (10,808× *g*, 10 min). Soluble proteins were precipitate by 80% ammonium sulfate and re-suspended in Tris-buffered saline (TBS) buffer, pH 7.4. Protein concentration was measured by Bradford assay, using Bio-Rad protein assay solution (Bio-Rad) [54].

### 4.9. Cellulose Hydrolysis Assay

A quantity of 0.6 mg/mL of *C. thermocellum* secretome solution was applied to a suspension of 250 mg/mL of microcrystalline cellulose in 20 mM citrate buffer, pH 6.1, with or without the addition of 2 µg/mL BglA (wild-type or mutant) in a reaction volume of 2 mL. Samples were incubated at 60 and 70 °C for 48 h, and centrifuged (16,100× *g*, 5 min). Released soluble sugars were analyzed by high-pressure liquid chromatography‏ (HPLC, Agilent Infinity 1260 system, Agilent Technologies, Santa Clara, CA, USA) using an Aminex^®^HPX-87H Ion Exclusion column (Bio-Rad, Hercules, CA, USA) with a guard column, mobile phase of 5 mM H_2_SO_4_ (flow-through of 0.6 mL/min at 45 °C) in an Agilent 1260 Infinity LC system with RID detector (G1362A). Experiments were performed in triplicate.

### 4.10. T_i_ Measurements

Recombinant BglA enzymes in TBSx1 buffer (with and without the addition of 1 mM cellobiose) were used for T_i_ measurements, using a NanoTemper Tycho NT.6 instrument (Agentek (1987) Ltd., Tel Aviv, Israel), according to the manufacturer’s instructions [42].

## Figures and Tables

**Table 1:** Kinetic parameters of *p*NPG hydrolysis by wild-type *C. thermocellum* BglA and the thermostable mutant (Mut 1).

Title	Wild Type	Mut 1
V_max_ [M·s^−1^]	9.92 × 10^−7^ ± 6.57 × 10^−8^	9.1 × 10^−7^ ± 3.97 × 10^−8^
K_cat_ [s^−1^]	76 ± 5.036	70 ± 3.05
K_m_ [mM]	6.7 ± 1.111	5 ± 0.59
K_cat_/K_m_ [s^−1^·M^−1^]	11,282 ± 900	14,018 ± 867

Kinetic parameters of *C. thermocellum* BglA and Mut 1 were measured by *p*NPG assay, and calculated by nonlinear fit by GraphPadPrism software.

## Supplementary Materials

Supplementary material can be found at https://www.mdpi.com/1422-0067/20/19/4701/s1.

## Abbreviations

BglA	β-glucosidase A
*p*NPG	*p*-nitrophenyl-β-d-glucopyranoside
CBM	cellulose binding module
