# Supplementary material for: Directed Evolution of Clostridium thermocellum β-Glucosidase A Towards Enhanced Thermostability
Source: Int J Mol Sci. 2019 Sep 23;20(19):4701. doi: 10.3390/ijms20194701 (PMC6801902; doi:10.3390/ijms20194701)
Supplement: Supplementary file 1 [file ijms-20-04701-s001.pdf]

Supplementary Material

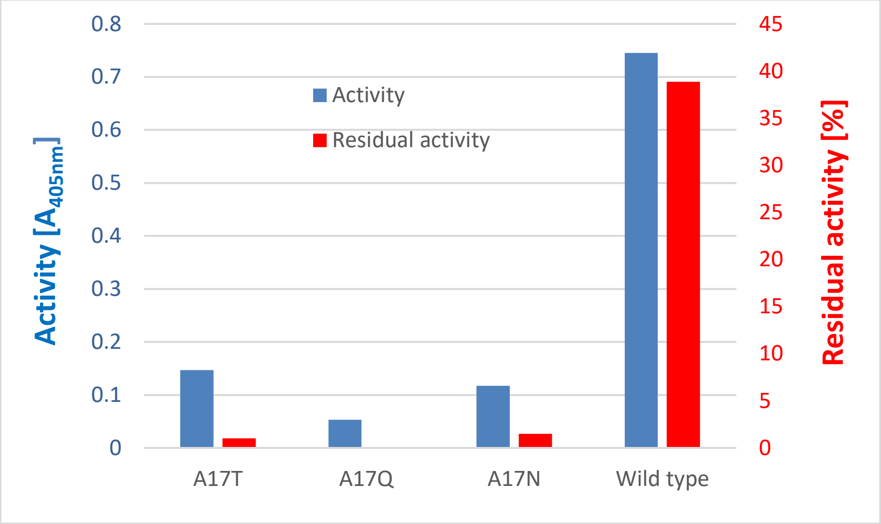

Figure 1. Residual activity of *C. thermocellum* BglA mutant enzymes. *E. coli* BL21 lysates containing either wild-type *C. thermocellum* BglA (Clo1313_2020) or one of the mutants (A17T, A17Q or A17N) were subjected to heat shock (66 °C for 75 min) followed by activity assay. Residual activity (activity of heat-shocked lysate·100/activity of non-heated lysate) was calculated.

3D Structure of *C. thermocellum* β-Glucosidase A (Clo1313_2020)

Crystallization of *C. thermocellum* BglA

Purified BglA (final concentration of 10 mg/ml) was a kind gift of CelDezyner (Rehovot, Israel). BglA was equilibrated against 25 mM Tris pH 7.0 and 25 mM NaCl. Initial crystallization screening trials using the Hampton Research Index kit showed several potential crystallization conditions. The best crystals were formed in condition #74 (0.1 M Bis Tris buffer pH 5.5, containing 0.2 M Lithium sulfate monohydrate and 25% w/v Polyethylene glycol 3,350). The crystal plates were obtained—approximately 14 d after plating, by the hanging drop vapor diffusion method at 18 °C.

X-ray data collection, processing and phasing of BglA

Crystals were removed to a cryoprotectant solution containing the mother liquor with 25% ethylene glycol and were stored in liquid nitrogen, until they were subjected to X-ray diffraction, using a synchrotron radiation source at beamline ID-23-A, ESRF, France. The dataset was indexed and scaled using HKL2000. BglA crystals belong to the monoclinic space group P21, with unit cell dimensions a = 91.66 Å, b = 56.24 Å, and c = 94.53 Å, β= 100.3°,

containing two molecules in the asymmetric unit. The crystal structure of BglA was solved using molecular replacement strategies, relying on the structure of 1QOX (53 % identity) [1] using AMoRe [2]. The structure was autotraced using ARP/wARP and refined by PHENIX [3] with diffraction data ranging from 30.00 to 1.65 Å resolution. The model was completed by manual corrections using COOT and refined to final crystallographic R_{cryst} and R_{free} values of 19.9%, and 23.1%, respectively (Table S1). BglA adopts the $(\beta/\alpha)_8$ TIM barrel fold. The 3D structure was published at the RSCB protein data bank (PDB code 5OGZ).

References

1. Hakulinen N, Paavilainen S, Korpela T, Rouvinen J. The Crystal Structure of β -Glucosidase from *Bacillus circulans* sp. *alkaliphilus*. J. Struct. Biol. 2000;79:69–79.

2. Navaza J. AMoRe: an automated package for molecular replacement. Acta Crystallogr. 1994;A 50:157–63.

3. Adams PD, Pavel V, Chen VB, Ian W, Echols N, Moriarty NW, et al. research papers PHENIX : a comprehensive Python-based system for macromolecular structure solution Acta Crystallogr. 2010;213–21.

Table S1: Data collection and refinement statistics.

	C. thermocellum BglA
Data Collection	
Beamline	ESRF ID-23-1
Wavelength (Å)	0.97300
Space Group	$P12_11$
Cell Dimensions	
a, b, c (Å)	91.67, 56.22, 94.50
Resolution (Å)	30-1.62 (1.64-1.62)
R_{sym} or R_{merge}	0.089 (0.406)
<I/σI>	20.7 (1.9)
Completeness (%)	94.61 (93.9)
Redundancy	5.0 (3.1)
No. of reflections	580841
No. Unique	120756
Refinement	
Resolution (Å)	1.60
R_{work}/R_{free}	0.18/0.21
No. of atoms	
Protein	3702 (A), 3702 (B)
Ligand	15 (SO4), 4 (EDO)
Water	479
B-factors	
Protein	17.19 (A), 21.52 (B)
Ligand	46.92 (SO4), 22.88 (EDO)
Water	27.12
r.m.s.d	
Bond lengths (Å)	0.02
Bond angles (°)	2.02
Ramachandran (%)	
Preferred	98
Generously allowed	15
Disallowed	1
PDB code	5OGZ
